# Do brain T2/FLAIR white matter hyperintensities correspond to myelin loss in normal aging? A radiologic-neuropathologic correlation study

**DOI:** 10.1186/2051-5960-1-14

**Published:** 2013-05-09

**Authors:** Sven Haller, Enikö Kövari, François R Herrmann, Victor Cuvinciuc, Ann-Marie Tomm, Gilbert B Zulian, Karl-Olof Lovblad, Panteleimon Giannakopoulos, Constantin Bouras

**Affiliations:** 1 Service neuro-diagnostique et neuro-interventionnel DISIM, University Hospitals of Geneva, rue Gabrielle Perret-Gentil 4,1211, Geneva 14, Switzerland; 2 Department of Mental Health and Psychiatry, Geneva, Switzerland; 3 Department of Internal Medicine, Rehabilitation and Geriatrics, University Hospitals of Geneva, Geneva, Switzerland; 4 Department of Readaptation and Palliative Medicine, University Hospitals of Geneva and Faculty of Medicine of the University of Geneva, Geneva, Switzerland

## Abstract

**Background:**

White matter hyperintensities (WMH) lesions on T2/FLAIR brain MRI are frequently seen in healthy elderly people. Whether these radiological lesions correspond to irreversible histological changes is still a matter of debate. We report the radiologic-histopathologic concordance between T2/FLAIR WMHs and neuropathologically confirmed demyelination in the periventricular, perivascular and deep white matter (WM) areas.

**Results:**

Inter-rater reliability was *substantial-almost perfect* between neuropathologists (kappa 0.71 - 0.79) and *fair-moderate* between radiologists (kappa 0.34 - 0.42). Discriminating low versus high lesion scores, radiologic compared to neuropathologic evaluation had sensitivity / specificity of 0.83 / 0.47 for periventricular and 0.44 / 0.88 for deep white matter lesions. T2/FLAIR WMHs overestimate neuropathologically confirmed demyelination in the periventricular (p < 0.001) areas but underestimates it in the deep WM (0 < 0.05). In a subset of 14 cases with prominent perivascular WMH, no corresponding demyelination was found in 12 cases.

**Conclusions:**

MRI T2/FLAIR overestimates periventricular and perivascular lesions compared to histopathologically confirmed demyelination. The relatively high concentration of interstitial water in the periventricular / perivascular regions due to increasing blood–brain-barrier permeability and plasma leakage in brain aging may evoke T2/FLAIR WMH despite relatively mild demyelination.

## Background

White matter hyperintensities (WMH) lesions on T2 and fluid attenuated inversion recovery (FLAIR) brain MRI are very common findings in elderly cohorts and their prevalence increases from 15% at the age of 60 to 80% at the age of 80 [1-4]. Mainly located in the periventricular white matter (WM) and perivascular spaces, they can also be detected in deep WM. Initially described in patients with cardiovascular risk factors and symptomatic cerebrovascular disease [4], WMHs are thought to have a deleterious effect on cognition and affect in old age (for review see [5-7]). Some studies indicate that periventricular but not deep WMHs affect neuropsychological performances [8-10] whereas other studies led to the opposite conclusion (for review [6]). In the same line, deep white matter and to a lesser degree periventricular hyperintensities are more common and more severe among individuals with late-onset depression than in healthy controls [11,12]. At the tissue level, WMH-associated damage ranges from slight disentanglement of the matrix, enlarged perivascular spaces due to lack of drainage of interstitial fluid and, in severe cases, irreversible myelin and axonal loss. Glial cell responses include astrogliosis and clasmatodendrosis as well as loss of oligodendrocytes and distinct microglial responses (for review see [13]). Among these lesions, degeneration of myelin is the most frequently encountered in old age and may take place long before the emergence of cognitive or affective symptoms [14]. Whether or not the frequent identification of T2/FLAIR WMHs in healthy elderly individuals represents an innocuous phenomenon or should be viewed as potentially harmful for brain structure is unknown. To address this issue, we performed a radiologic-histopathologic correlation analysis of T2/FLAIR WMHs in periventricular and perivascular regions as well as deep WM in elderly subjects, who had brain autopsies and pre-mortem brain MRIs. Since the T2/FLAIR signal depends on the local concentration of water in interstitial spaces, we postulated that the sensitivity and specificity values for WMHs might depend on the anatomic location studied. We tested the hypothesis that periventricular WMHs might overestimate demyelination given the relatively high local concentration of water in this brain area.

## Methods

### Selection of cases

The local ethical committee approved this retrospective study. All cases were drawn from the brain collection of the Geriatric Hospitals of Geneva County. During a 10-year period from 1.1.2000 and 31.12.2010, 1064 cases were autopsied in this hospital as part of a systemic procedure in an academic geriatric hospital. All of the patients were neuropsychologically evaluated using a Mini-Mental State Examination [15] performed at least once during the last month prior to their death. 134 cases had a pre-mortem brain MRI on the local radiological database. Cases with clinically overt neurological diseases including stroke, Parkinson’s disease and other neurodegenerative conditions, cognitive disorders (including all forms of dementia and mild cognitive impairment), normal pressure hydrocephalus, chronic subdural hematoma, extra-axial masses as well as primary or secondary brain tumors and significant neurological symptoms prior to death (75 cases) were excluded from this study. The remaining 59 caucasian patients (32 women, mean age: 82.7 ± 6.7, 27 men, mean age: 80.5 ± 9.5) had MMSE scores between 28 and 30 and displayed various degrees of T2w lesions within the normal limits for their age. The mean delay between MRI scans and autopsy was of 5.4 ± 2.2 years (range: 0.1-11.4 years). Among cardiovascular risk factors hypertension was present in 33 (55.9%), hypotension in 11 (18.6), dyslipidemia in 10 (17.2) and diabetes in 12 (20.3%) subjects of the sample. Cause of death were 30 (50.9%) bronchopneumonia, 9 (15.3%) cancer, 7 (11.9%) cardiovascular, 5 (8.5%) sepsis, 3 (5.1%) pulmonary emboli, 2 (3.4%) brain hemorrhagia and 3 others.

The neuropathological examination of these 59 cases revealed no silent brain infarcts or other macroscopic alterations as tumors or inflammation. Consistent with the very old age of our cohort [16], three cases showed Braak stages 5 for neurofibrillary tangles [17] and 8 cases had at least one cortical Lewy body [18]. Only two cases showed severe amyloid angiopathy. No other histological lesions potentially associated with WM lesions were observed. An ependymal denudation of variable extension (at least of microscopic size) was present in all cases on the ventricular surface.

### Radiologic and pathologic analysis

Three trained neuroradiologists evaluated brain T2w and FLAIR MRI of all 59 cases blind to the neuropathologic data. All included cases had axial spin-echo T2 and coronal FLAIR imaging. Due to the period of 10 years, the exact MRI parameters varied. Slice thickness of axial T2W and coronal FLAIR ranged between 3 and 4 mm. The severity of WMHs was estimated using an adapted version of the widely used Fazekas semiquantitative rating scale for periventricular and deep WMHs [19]. The periventricular WMHs were defined as T2/FLAIR signal alterations in direct contact with the ventricular system. The deep WMHs were defined as T2/FLAIR signal alterations distant from the ventricular system. Periventricular and deep white matter WHMs could co-exist. Periventricular WMHs were scored as follows: 0, absent; 1, pencil lines and/or caps; 2, smooth haloes; and 3, irregular. Deep WMHs were scored as follows: 0, absent; 1, punctate; 2, coalescing; and 3, confluent. Additionally, axial T1w, T1w after Gadolinium administration and T2*w images were analyzed to rule out concomitant brain pathological findings.

**Figure 1:**
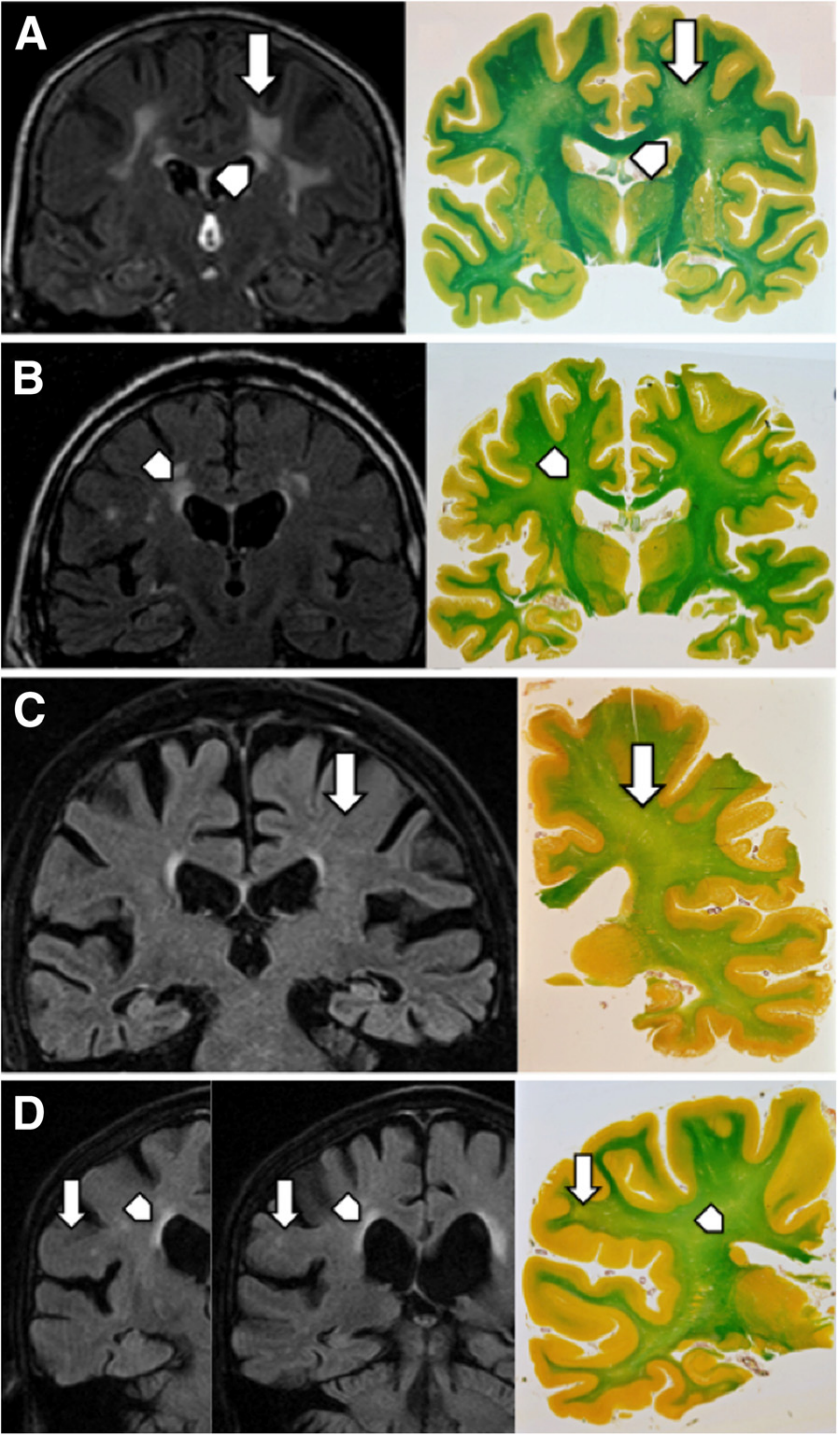
**Representative examples of the concordance between brain MRI WMHs and demyelination. (A)** Good correlation between radiology and pathology for both periventricular (arrowhead) and deep WM (arrow) lesions; **(B)** radiological assessment over-estimating periventricular lesions; **(C)** under-estimating deep WM lesions; **(D)** over-estimating periventricular lesions and under-estimating deep WM lesions. Radiologic convention, right hemisphere on left hand side. Coronal fluid attenuated inversion recovery (FLAIR) image and corresponding histophatologic slice in Luxol-van Gieson staining with normal WM in green and regions of demyelination in faint green-yellow.

The neuropathological assessment was performed prospectively on the basis of MRI findings. Whole coronal brain slices were taken corresponding to the level (three slides/level) where WMHs were most pronounced. In the absence of T2w lesions slices (n = 3) at the level of the lateral geniculate nucleus were examined. Coronal slice orientation during analysis was the same for radiology and neuropathology. From paraffin-embedded blocs 2 consecutive 12 μm thick slides were cut and stained with Luxol-van Gieson staining for the visualization of myelin as well as haematoxylin-eosin and haematoxylin-eosin for cellular and structural analysis [20]. Histological slides were independently evaluated by two trained neuropathologists without previous knowledge of the MRI data. According to Scheltens et al. [21], the severity of periventricular and deep WM demyelination was assessed on a 4-level semi-quantitative scale, where 0 corresponded to absent; 1 to mild; 2 to moderate and 3 to severe demyelination. As already indicated in this early report, the severity of periventricular and deep WMdemyelination closely correlates with its extent (Figure 1).

**Figure 2:**
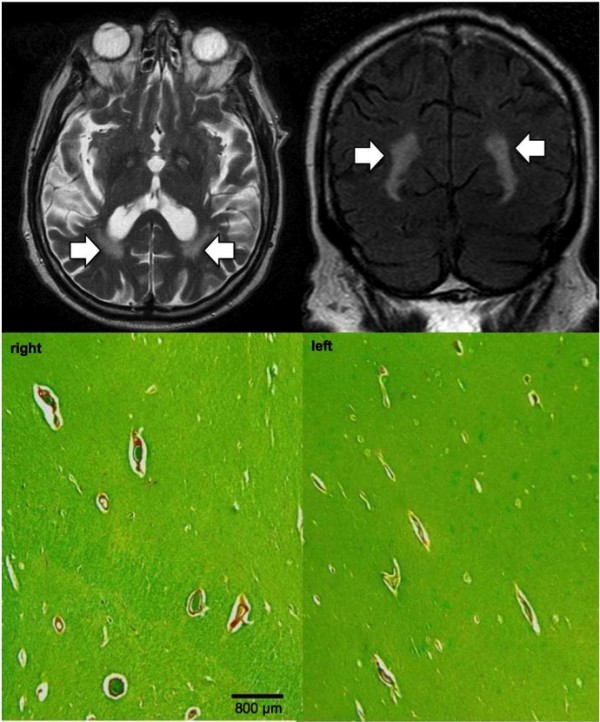
**Prominent perivascular spaces evident as radial linear hyperintesities on T2 with additional perivascular confluent WMH in bilateral temporo-occipital WM (A axial T2, B coronal FLAIR).** The corresponding histopathology confirms the presence of prominent perivascular spaces, yet there is no significant demyelination around the perivascular spaces, which would correspond to the confluent hyperintense T2/FLAIR signal alteration. Scale bar = 800 micrometers.

We also identified a subset of 14 cases in the whole series that displayed prominent T2/FLAIR WMHs around perivascular spaces on brain MRI defined as confluent T2/FLAIR lesion immediately adjacent to prominent and clearly visible perivascular spaces on T2w (see Figure 2). These lesions were typically located in the parietal lobes between periventricular and deep white matter. The corresponding Luxol-van Gieson (LVG)-stained histological slides were analyzed by both pathologists assessing the degree of demyelination around the perivascular spaces.

### Statistical analysis

#### Assessment of inter-rater agreement

We computed average scores within each group and then dichotomized the averaged scores using a threshold of 1.5. The threshold of 1.5 corresponds to the rounding of the scores to the nearest integer values. Thus a threshold below 1.5 corresponds to rounded value of 0 and 1 (low lesion load) and above or equal to 1.5, corresponding to scores of 2 or 3 (high lesion load). We opted for this method in order to avoid that similar yet not identical categories would be classified as mismatch. However, this statistical approach may overestimate the concordance values in the present study. These values are then illustrated in 2 x 2 tables (see Table 1).

**Table 1:** Two by two tables comparing the neuropathologists vs radiologists averaged dichotomized scores for periventricular and deep WM lesions, along with the kappa statistics, the Mc Nemar test to compare discordant pair (shown as cells with darker background), sensitivity and specificity values (neuropathology as gold standard)

		**Radiologist**			**Sensitivity**			**Specificity**		
		**Score < 1.5**	**Score ≥ 1.5**	**Total**	**Estimate**	**95% CI**		**Estimate**	**95% CI**	
**Periventricular demyelination**										
** Pathologist**	Score < 1.5	25	**28**	53	0.83	0.36	1.00	0.47	0.33	0.61
	Score ≥ 1.5	**1**	5	6						
	Total	26	33	59						
**Deep WM demyelination**										
** Pathologist**	Score < 1.5	30	**4**	34	0.44	0.24	0.65	0.88	0.73	0.97
	Score ≥ 1.5	**14**	11	25						
	Total	44	15	59						

In a first step, we assessed the inter-rater agreement using kappa statistics presented with 95% confidence interval (95% CI). For neuropathologists (2 raters) we used standard Cohen’s kappa testing. For radiologists (3 raters) we used binary ratings. 95% confidence interval (CI) for the kappa statistics were calculated using bootstrap with 1000 replications. Z-tests were used to compare kappa with zero. Landis and Koch's interpretations of kappa were used as follows [22]:< 0.0 Poor, 0.00 – 0.20 Slight, 0.21 – 0.40 Fair, 0.41 – 0.60 Moderate, 0.61 – 0.80 Substantial, 0.81 – 1.00 Almost perfect.

In order to explore whether a simple qualitative approach improves the inter-rater agreement, the same analysis was performed for the presence/absence of lesions. Kappa statistics were also repeated with a subsample of 33 cases with delay between MRI and autopsy less than 5 years (median delay (interquartile range, IQR): 4.2 (0.4), mean ± standard deviation 4.0 ± 1.1 years).

### Discordance between radiologic and pathologic scores

Discordant pairs were analyzed with exact Mc Nemar significance probability. This procedure tests the null hypothesis that the probability of each discordant pair (the cells of a 2 by 2 tables which are not over the diagonal) is equal versus the opposite. In the latter case, the result is interpreted as a significant over- or under-estimation. Again, all tests were repeated with a subsample of 33 cases with delay between MRI and autopsy less than 5 years.

### Multiple regression analyses adjusting for demographic data and MRI-autopsy delay

Finally, we assessed the effects of other clinical parameters using multiple linear regression models with the pathological score as the dependent variable and radiological score, age, sex, and delay between MRI and death as the independent variables. T-tests were used to compare regression coefficients with zero. P values inferior to 0.05 were considered significant. The coefficient of determination (R^2^) was used to assess the proportion of variance explained by the models. All statistics were performed with Stata release 12.1, Stata Corp., College Station, TX, USA 2012 (FRH 21 years of experience).

## Results

### Inter-rater agreement (N = 59)

The agreement between neuropathologists was substantial both for periventricular (kappa of 0.71 (95% CI: 0.53 - 0.87; p < 0.0001)) and deep WM demyelination (kappa of 0.79 (95% CI: 0.65 - 0.93; p < 0.0001)). In contrast, radiologists showed moderate agreement for periventricular WMHs (kappa of 0.42 (95% CI: 0.31-0.55; p < 0.0001)) and only fair agreement for deep WMHs (kappa of 0.34, 95% CI: 0.22-0.48; p < 0.0001)). There was a slight agreement between neuropathologists and radiologists for periventricular lesions with kappa value of 0.10 (95% CI: -0.03 - 0.23; p = 0.077). A fair agreement between neuropathologists and radiologists was observed for deep WM lesions with kappa value of 0.34 (95% CI: 0.11 - 0.57; p = 0.003). Importantly, when the presence/absence of lesions was considered, kappa values did not change significantly for neuropathologists (0.74/95% CI:0.58-0.89 for periventricular and 0.65/95% CI: 0.28-0.99 for deep WM demyelination), improved for radiologists (0.57/95% CI: 0.37-078 for periventricular and 0.50/95% CI: 0.31-0.70 for deep WMHs) but became even worse for radiologic-pathologic correlations (−0.05/95% CI:-0.11-0.01 for periventricular and 0.12/95% CI:-0.20-0.43 for deep WM lesions).

In 28 cases, radiologists made an overestimation of lesion scores for periventricular demyelination (Table 1). Only in one case, they underestimated the underlying pathology (exact McNemar p < 0.001). Compared to the neuropathologic reference standard, radiological assessment for periventricular WMHs showed a good sensitivity (83%) but only low specificity (47%) (Table 1). In multiple linear regression models, the only variable significantly associated with the neuropathologic score was the radiological score (regression coefficient 0.21; 95% CI: 0.04-0.38; p = 0.019) that explained 15% of its variance. The other independent variables were not related to the neuropathological score.

In contrast to periventricular lesions, radiologists only rarely overestimated deep WM lesions (4 cases) but underestimated it in 14 cases (Exact McNemar p = 0.031). Sensitivity value for radiological cut-off was modest at 44% but specificity was good at 88% (Table 1). In multiple linear regression models, only the radiological score predicted the neuropathologic score (regression coefficient of 0.29; 95% CI: 0.06-0.52; p = 0.016) explaining 22% of its variance (Figure 1).

No explicit astrocytosis or clasmatodendrosis was present in the haematoxylin-eosin-stained slides.

### Subsample of subjects with delay < 5 years between MRI and autopsy

Age (79.7 ± 8.9 vs 81.6 ±10.2, p = 0.4686) and gender (male 14 (42.4%) vs 13 (50.0%), p = 0.607) distribution were not significant different between patients with a delay below 5 or ≥5 years, respectively. The agreement between neuropathologists was substantial both for periventricular (kappa of 0.65; 95% CI: 0.60 - 0.85; p < 0.0001) and deep WM demyelination (kappa of 0.78; 95% CI: 0.59 - 0.95; p < 0.0001)). In contrast, radiologists showed fair agreement for both periventricular WMHs (kappa of 0.38; 95% CI: 0.22 - 0.55; p < 0.001)) and for deep WMHs (kappa of 0.32; 95% CI: 0.16 – 0.49; p < 0.001). There was a fair agreement between neuropathologists and radiologists for periventricular lesions with kappa value of 0.31 (95% CI: -0.03 - 0.59; p = 0.023). Radiologists overestimated these lesions in 16 cases. In no cases did they underestimate the underlying pathology (exact McNemar p < 0.001). Sensitivity value for radiological cut-off was excellent at 100% (95% CI: 48% - 100%) but specificity was modest at 43% (95% CI: 25% - 63%). A slight agreement between neuropathologists and radiologists was observed for deep WM lesions with kappa value of 0.19 (95% CI: 0.02 - 0.35; p = 0.033). In contrast to periventricular lesions, radiologists overestimated the pathology only in 3 cases and underestimated it in 10 cases (exact McNemar: p = 0.092). Sensitivity value for radiological cut-off was 38% (95% CI: 15% - 64%) but specificity reached 82% (95% CI: 57% - 96%).

### Analysis of WMHs around perivascular spaces on MRI

In 12 among the 14 cases with prominent perivascular WMHs, histopathologic demyelination of the region around the Virchow-Robin spaces was absent (Figure 2). Demyelination of the perivascular WM was seen only in 2 cases (14.3%), as a part of a severe global demyelination.

The presence of hypertension, hypotension, dyslipidemia or diabetes was not associated with agreement between radiologist or pathologist in logistic regression models predicting agreement.

## Discussion

The present study revealed that brain T2/FLAIR sequence-identified WMHs overestimated demyelination in the periventricular and perivascular regions but underestimated it in the deep WM during normal brain aging. We suggest that a possible explanation of this dissociation may reside in the differences in local concentration of interstitial water between these brain areas. In fact, previous investigations suggested increasing leakage of plasma into the WM [23-25] and increased blood–brain-barrier permeability [25] during aging, inducing a relatively high local water concentration in the periventricular and perivascular regions. Consequently, a relatively low degree of histopathologically documented demyelination may be sufficient to induce T2/FLAIR signal alterations. In contrast, due to the relatively low local water concentration in the deep WM, a relatively higher degree of demyelination might be necessary to induce the same amount of T2/FLAIR signal abnormality.

Previous radio-pathological studies on WMHs are very rare. A recent review of post-mortem MRI in patients with small vessel disease pointed to the marked heterogeneity of the pathologic correlates of WMHs [13]. In old age, WMHs were mainly associated with myelin pallor, tissue rarefaction including loss of myelin and axons, and mild gliosis [3,23,26-28]. The only radio-pathological study with pre-mortem MRI included only 23 unselected cases and reported that vascular integrity was the only parameter that correlated with total WMH [29]. The present study is based on a larger sample of carefully selected cases with preserved cognition. The severity of demyelination in postmortem tissue was positively associated with the WMH lesion score both in periventricular and deep WM areas. However, this association remained modest since radiological scores explained only 15 to 22% of the variability in pathological scores. Importantly, this weak association was obtained despite the use of a simple semi-quantitative scale that was expected to increase the agreement between neuropathologists and radiologists. The inclusion of computer assisted data analysis such as machine-learning derived support vector machine analyses may allow for detecting subtle changes, which are not reliably detected by visual inspection [30,31]. Multimodal data acquisition going beyond classic T2/FLAIR imaging including diffusion tensor imaging (DTI) to assess WM microstructure [32,33] and magnetization transfer imaging (MT) [34] to discriminate “free” versus “restricted” or bound water compartments may also contribute to improve the radio-pathologic correlations.

The clinical significance of WMHs in healthy controls remains controversial. All of the cases included in the present series presented with high MMSE scores compatible with normal cognitive functioning and absence of major depression. The presence of demyelination was mild to moderate in most cases in periventricular and deep WM. It is thus likely that the severity of histopathological changes was not sufficient to affect cognition and emotional regulation in these very old individuals. Two recent studies in healthy controls indicated that WMHs are associated with subtle executive dysfunctions and reduced speed of information processing [35,36]. Another study revealed that severe white subcortical WMHs (odds ratio 5.4) were more likely to have depressive symptoms compared to periventricular matter lesions (odds ratio 3.3) [37]. In the same line, another cohort study supported the clinical relevance of deep WMHs that were correlated with cardiac arrhythmia, brain atrophy, and silent infarcts [2]. The present results indicate that the systematic detection of periventricular WMHs in old age should be viewed with caution since they may correspond to innocuous histological changes. In contrast, deep WMHs should be considered as an in situ pathology and not a simple epiphenomenon of brain aging.

The main strength of the present study is the unusually large autopsy series of very old healthy controls with MRI documentation. Other strengths include separate assessment of periventricular, deep WM and perivascular pathology, and the use of multivariate models controlling for MRI-autopsy delay. However, several limitations should also be considered when interpreting our data. One main caveat to consider is the relatively long MRI-autopsy delay in this study. Assuming that brain MRI WMHs are irreversible, this delay is not relevant with respect to the overestimation of pathology by MRI T2/FLAIR scans in periventricular areas. However, one could argue that the underestimation of demyelinating lesions in deep WM may be due to the formation of new lesions during the variable delay between MRI and autopsy. Although there is no clear consensus about the age-related evolution of WMH, recently accumulated data suggested that elderly individuals with punctuate abnormalities have a low tendency for progression compared to those with early confluent changes (see [38]). In community-based series, the volume of WMH in these latter cases increases by as much as one quarter per year. The additional analysis in a sub-sample of 33 cases with an MRI-autopsy delay inferior or equal to 5 years led to similar results. Most importantly, in multivariate models, the MRI-autopsy delay had no significant impact on the association between radiological and neuropathologic scores. As is usually the case for neuropathologic analyses, the retrospective design represents an additional limitation of our study. Although all of the cases had no major cognitive deficits and clinically overt depression, we cannot exclude the presence of subtle neuropsychological deficits or subsyndromal depression that may be related to WMHs. Another limitation concerns certain a priori choices in respect to the radiological and neuropathological investigations. We analyzed the pathological significance of T2/FLAIR sequences since they are the most widely available in routine clinical settings. However, they are suboptimal to detect the whole range of WMHs and microstructural changes in old age. In particular, abnormalities in crossing fibers that may be identified by diffusion tensor imaging (DTI) sequences may partly explain the development of WMH in this age group. As expected, slice thickness was very different in MRI compared to neuropathological analysis. We cannot thus formally rule out a partial volume effect on MRI. Moreover, the use of automatic segmentation analyses of WMHs and quantitative assessment of demyelination in postmortem material is certainly more reliable for exploring the association between radiological observations and neuropathologic findings. Finally, this study focused on demyelination as main histopathologic lesion. In the absence of unbiased histological methods, we cannot demonstrate the relatively high local water content, which might be one potential origin for the hyperintense T2/FLAIR signal in periventricular areas as discussed above. One should however note that denudation of the ependymal layer was present in all of our cases, which might facilitate plasma leakage in the periventricular region.

Prospective studies in elderly cohorts with minimal MRI-autopsy delay including DTI and MT sequences, assessment of the glial pathology associated with WMHs and quantitative radio-pathological evaluation are warranted to clarify the significance of WMHs in the course of brain aging.

## Conclusions

MRI T2/FLAIR overestimates periventricular and perivascular brain lesions during normal aging compared to histopathologically confirmed demyelination. The relatively high concentration of interstitial water in the periventricular / perivascular regions in combinations with the increasing blood–brain-barrier permeability and plasma leakage in brain aging may contribute to T2/FLAIR WMH despite relatively mild demyelination.

## Abbreviations

DTI: Diffusion tensor imaging; FLAIR: Fluid attenuated inversion recovery; MMSE: Mini mental score examination; MRI: Magnetic resonance imaging; MT: Magnetization transfer (imaging); WM: White matter; WMH: White matter hyperintensities.

## Competing interests

The author declares that they have no competing interests.

## Authors’ contributions

SH, K-OL, EK, and CB designed the study. SH, VC, and A-MT did radiological evaluation. EK and CB did data collection and histological analyses. FRH performed statistical analyses. All authors participated in the data interpretation. SH, EK and PG wrote the paper. EK, CB and PG provided critical reading of the manuscript. All authors approved the final version of the manuscript.
